# Stroke in the Very Old: A Systematic Review of Studies on Incidence, Outcome, and Resource Use

**DOI:** 10.4061/2011/108785

**Published:** 2011-08-16

**Authors:** Tommasina Russo, Giorgio Felzani, Carmine Marini

**Affiliations:** Dipartimento di Medicina Interna e Sanità Pubblica, Università degli Studi di L'Aquila, 67010 Coppito, Italy

## Abstract

*Background and Purpose*. Stroke incidence increases with age and is likely to increase in the aging populations. We investigated incidence, outcome, and resource use in very old subjects with stroke. *Methods*. We performed a systematic review of available data through electronic search of the literature databases and manual search of reference lists. Data were extracted for the age groups of over 80, 80 to 84 years old, and over 85. Overall incidence rates, expressed as the number of first strokes per 1000 person-years, were estimated using Poisson regression analysis. Odds ratios for the comparisons between subjects over and under 80 were calculated with the Mantel-Haenszel method. *Results*. We found a high incidence of stroke in the very old. The estimated incidence rates were 20.78 (95% CI 19.69 to 21.87) in subjects over 80, 17.23 (95% CI 15.97 to 18.49) for those 80 to 85 years old, and 20.78 (95% CI 16.74 to 23.78) for those over 85. Subjects over 80 contributed 29.95% of strokes; rates were similar among genders. Thirty-day case fatality rate and occurrence of dependency were higher in subjects over 80, although associated with less frequent hospital and stroke unit admission and less diagnostic resource use. *Conclusions*. The contribution of very old subjects to the global burden of stroke is relevant and may require efficient dedicated stroke services.

## 1. Introduction

In many Western countries, subjects in the oldest age classes, usually referred as the oldest old or very old, represent the fastest to growing segment of the population and make a huge contribution to health care costs [1]. Stroke is one of the leading causes of death and of severe disability in most countries, and its incidence increases steeply with age [2]. Thus, in the forthcoming years, stroke may represent a massive epidemic, causing many disabled patients and deaths in Western countries [2–5]. The availability of data on incidence, classification, and prognosis of stroke in the very old and information on resource use is important to plan health services and to focus treatment strategies. However, studies in the very old are sparse, small, and differing in methodology [4, 6–35].

We performed a systematic review of the available evidence on incidence, outcome, and resource use of very old people with stroke.

## 2. Materials and Methods

In the present paper, data were identified by search of Medline and from the references of relevant articles published after 1980. Different subsets of studies were potentially eligible for different parts of this paper. The search terms “stroke”, “isch(a) emic stroke”, “intracerebral”, “intraparenchymal”, “subarachnoid”, “h(a) emorrhage” were firstly used. Then the search was refined by applying any of the following terms: “population-based”, “community-based”, “community”, “epidemiology”, “epidemiological”, “incidence”, “occurrence”, “survey”, “surveillance”, “prognosis”, “outcome”, “management”, and “resource use”. Lastly the terms “very old”, “oldest old”, “very elderly”, and “over 80” were applied for the final search refinement. Only papers published in English were reviewed. The reference list of the identified papers were also manually searched. Stroke had to be defined according to the WHO definition, that is, the occurrence of rapidly developing signs of focal or global disturbance of cerebral function, lasting longer than 24 hours or leading to death, with no apparent cause other than that of vascular origin [36].

Two of the authors reviewed all selected papers reporting data on occurrence, management, and outcome of stroke in subjects over 80 years of age, 80 to 85 years old, or over 85 years. Data on absolute and relative incidence of stroke, stroke type and demographics, outcome, diagnostic procedures, and treatment were assessed. Any repeated reporting of the same study was excluded, so that each data set was considered only once. Population-based studies performed in different period on the same population were considered only once, by using the final data assessment. Stroke type classification was considered only in those studies where CT, MRI, or autopsy findings were available for at least 80% of stroke cases. Strokes were classified into four major types: ischemic stroke (if CT or MRI within 30 days of stroke showed infarct or no relevant lesion and/or autopsy showed ischemic stroke), primary intracerebral haemorrhage (if shown on CT, MRI, or autopsy), subarachnoid haemorrhage (classified by characteristic findings in CSF analysis and/or autopsy, CT, or cerebral angiography), and undetermined stroke (no CT, MRI, autopsy, cerebral angiography, or (for subarachnoid haemorrhage only) CSF examination was done). 

The incidence of first ever stroke was calculated per 1,000 person-years. Poisson regression analysis was used to compare incidence rates from different studies. Fitted values were assumed as the best estimate of the true stroke incidence in the very old. Odds ratios with 95% confidence intervals (95% CI) were calculated for mortality, dependency at the modified Rankin scale (mRS), and healthcare resource use with the Mantel-Haenszel method. Sensitivity analysis was performed by excluding those studies that produced a significant deviance change when removed from the model. Since only few studies used the classical cutoff of age 80, results were presented separately for subjects over 80, 80 to 84 years old, and over 85.

## 3. Results

Sixteen studies reporting data on stroke incidence in the very old were identified including altogether 2406 patients 80 years old or older with a stroke occurring over 114,074 person-years at risk. Incidence rates and confidence intervals are reported in Table 1. Only two studies reported incidence data in subjects over 80 (estimated overall incidence rate of 20.78 per 1,000 person-year; 95% CI 19.69 to 21.87), and three studies reported incidence in people aged 80 to 84 years (overall incidence 17.23/1,000; 95% CI 15.97 to 18.49). In both analyses, there was a significant heterogeneity (*P* < 0.0001). Incidence in people over 85 was reported by 15 studies, with a significant variability among studies (*P* < 0.0001) and rates ranging between 10.34 and 33.48 per 1,000 person-year; the estimated overall incidence rate was 20.78/1000; 95% CI 16.74 to 23.78.

Incidence was almost similar between men and women in the considered age classes of very old people (Table 1). About one-third (29.95%) of strokes occurred in subjects over 80, 15.21% between 80 and 84 years of age, and 16.78% in those over 85.

The distribution of stroke type was reported by 6 studies (Table 2). The estimated overall occurrence rates indicated that the great majority of subjects suffered from an ischemic stroke (88.27%); intracerebral hemorrhage occurred in a proportion of subjects (11.17%) similar to that of all ages subjects (13.43), while subarachnoid hemorrhage was quite rare in the very old (0.55%).

**Figure 1:**
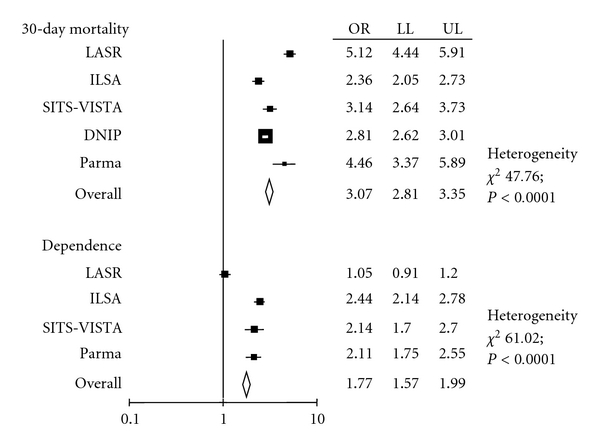
Meta-analysis of studies on stroke outcome in the very old. LASR: L'Aquila stroke registry; ILSA: Italian longitudinal study on aging; SITS-VISTA: International Stroke Thrombolysis Registry and Virtual International Stroke Trials Archive; OR: odds ratio; LL: lower limit; UL: upper limit.

Stroke outcome in oldest subjects was reported by two population-based and three hospital-based studies. Thirty-day case fatality rates and the proportion of dependent subjects (modified Rankin scale > 2) were reported in Figure 1. Mortality was consistently higher among subjects over 80 as compared to subjects under 80 (OR 3.07; 95% CI 2.81 to 3.35). The proportion of dependent subjects was also significantly higher among subjects over 80 in all studies, but in the L'Aquila stroke registry, with an overall OR of 1.77 (95% CI 1.57 to 1.99).

**Figure 2:**
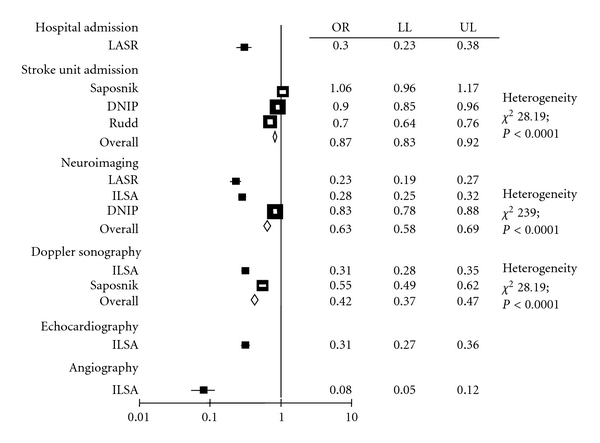
Meta-analysis of studies on healthcare resource use by very old people. LASR: L'Aquila stroke registry; ILSA: Italian longitudinal study on aging; DNIP: Danish National Indicator Project; OR: odds ratio; LL: lower limit; UL: upper limit.

Sparse data are available on resource use by very old subjects with acute stroke (Figure 2). However, in the majority of studies, there was a general tendency to lower use of healthcare resources in subjects over 80, with a tendency to less frequent hospital and stroke unit admission and less frequent neuroimaging Doppler sonographic, echocardiographic, and angiographic studies.

## 4. Discussion

Data on stroke incidence, clinical and demographic characteristics, and healthcare resource use in the very old are scarce and often inconsistent. The age cut-off of 80 years was close to the average life expectancy in many Western countries and was crucial, considering the sharp drop in the general population after that age [1, 34]. However, only a few studies reported incidence of stroke in patients over 80. On the other hand, there were only moderate differences in the incidence rates among subjects 80 to 84 years old and those over 85. In fact, incidence of stroke was very high in subjects 80 to 84 years old (17.23/1,000) as well as in those over 85 (20.78/1,000).

There is a considerable heterogeneity among incidence rates in different studies. The lowest rate was reported by the study performed in Basle where death certificates were not directly searched [20]. Though lifestyle characteristics of the population and risk factors control might have influenced incidence in the elderly in different studies, rising important clues on the implementation of preventive measures, the play of competing risks in subjects over 85 and of end-cohort effects might also explain most of the differences.

As strongly recommended by several authors, we analyzed incidence of first-ever stroke only, since the inclusion of subsequent strokes, occurring in a highly selected population of stroke survivors, may produce highly biased results [37]. However, in order to estimate the true occurrence of any stroke, either first or recurrent, a 30% of events should be added, producing even more impressing incidence rates in the very old [17].

According to our estimates, subjects over 80 years of age contributed almost one third of all strokes pointing out that this small segment of the resident populations makes a relevant contribution to the global burden of stroke. Moreover, although stroke incidence is similar in very old men and women, because of the higher proportions of women in these age classes, women are likely to be responsible for an increasing number of stroke admissions in many countries with a rapidly aging population [1–3, 5, 34].

Besides, despite 30-day case fatality rate was much higher in subjects over 80 than in younger subjects (OR 3.07), surviving patients still showed a higher risk of dependency after stroke (OR 1.77). Therefore, very old people also contribute to stroke costs that are likely to increase as general populations get older. 

In the available studies, there is a clear tendency to a lower resource use, balancing the tendency to higher costs of stroke in the very old [8, 19, 30, 32, 33]. This tendency may depend on different attitudes and traditions in health care utilization leading to inequalities in the access to health care resources, rather than on fewer requirements and may rise some ethical concern unless adequately prevented. Ad hoc services and dedicated access routes may be useful to reduce inequalities in resource use and to improve health services for the very old.

In conclusion, stroke in the very old is a very frequent condition with an unfavourable outcome, make a relevant contribution to the social burden of stroke, and may require more efficient, dedicated stroke services.

## Figures and Tables

**Table 1:** Studies on incidence of stroke in the elderly.

Study	Rate ∗ 1000	95% CI	M/F	% very old
Age over 80 years				
LASR	21.54	20.39*–*22.69	1.04	30.23
Dijon	10.68	7.71*–*13.65	0.99	23.90
*Overall *	*20.78*	19.69*–*21.87	*1.03*	*29.95*

Heterogeneity *χ* ^2^ = 24.23; *P* < 0.0001				

Age 80–84 years				
LASR	17.41	16.11*–*18.71	1.09	15.46
Dijon	8.87	5.26*–*12.48	1.32	11.22
ILSA	14.38	9.62*–*19.15	1.13	14.11
*Overall *	*17.23*	15.97*–*18.49	*1.15*	*15.21*

Heterogeneity *χ* ^2^ = 11.42; *P* < 0.0001				

Age over 85 years				
LASR	30.00	27.71*–*32.28	1.21	14.77
Basle	10.34	7.76*–*12.92	1.22	22.68
Rochester	23.51	19.28*–*27.74	0.61	23.39
Auckland 1991	19.13	15.91*–*22.35	0.62	17.34
Dijon	13.03	8.05*–*18.00	0.74	12.68
Innherred	30.39	30.28*–*30.51	1.16	21.06
London	18.93	13.63*–*24.23	1.01	21.33
Perth	23.89	17.87*–*29.92	1.39	15.95
Belluno	33.48	26.17*–*40.78	0.68	16.46
Oxfordshire	19.87	15.78*–*23.95	0.90	13.19
Aosta	32.37	22.37*–*42.36	1.81	15.35
Frederiksberg	15.99	11.41*–*20.58	1.36	17.56
Umbria	21.80	15.82*–*27.77	0.75	17.30
ESPro	21.17	16.19*–*26.15	1.20	19.21
Arcadia	26.61	22.06*–*31.16	1.51	23.06
*Overall *	*20.78*	16.74*–*23.78	*1.07*	*16.78*

Heterogeneity *χ* ^2^ = 24.2; *P* < 0.0001				

**Table 2:** Distribution of stroke type in the very old.

Study	IS		ICH		SAH	
	%	95% CI	%	95% CI	%	95% CI
LASR	86.89	84.46*–*89.32	12.70	10.3*–*15.1	0.41	0.00–.86
Innherred	90.63	83.48*–*97.77	7.81	1.24*–*14.39	1.56	0.00–4.6
Belluno	94.03	88.36*–*99.7	5.97	.3*–*11.64	0.00	—
Frederiksberg	96.97	91.12*–*100	3.03	0.00*–*8.88	0.00	—
ESPro	96.43	91.57*–*100	3.57	0.00*–*8.43	0.00	—
Arcadia	86.18	80.08*–*92.28	12.20	6.41*–*17.98	1.63	0.00-3.86
*Overall*	*88.27*	*86.36–90.19*	*11.17*	*9.3–13.05*	*0.55*	*.11–1.00*

Heterogeneity *χ* ^2^ = 1.06; *P* = 0.9998						

*Overall all ages*	*83.75*		*13.43*		*2.82*	
